# Effects of sliding window variation in the performance of acceleration-based human activity recognition using deep learning models

**DOI:** 10.7717/peerj-cs.1052

**Published:** 2022-08-08

**Authors:** Milagros Jaén-Vargas, Karla Miriam Reyes Leiva, Francisco Fernandes, Sérgio Barroso Gonçalves, Miguel Tavares Silva, Daniel Simões Lopes, José Javier Serrano Olmedo

**Affiliations:** 1 Bioinstrumentation and Nanomedicine Laboratory, Center for Biomedical Technology, Universidad Politécnica de Madrid, Madrid, Spain; 2 Engineering Faculty, Universidad Tecnológica Centroamericana, San Pedro Sula, Honduras; 3 INESC ID, Lisbon, Portugal; 4 IDMEC, Instituto Superior Técnico, Universidade de Lisboa, Lisbon, Portugal; 5 Instituto Superior Técnico, Universidade de Lisboa, Lisbon, Portugal; 6 CIBER-BBN, Centro de Investigación Biomédica en Red en Bioingeniería, Biomateriales y Nanomedicina, Madrid, Spain

**Keywords:** Accelerometer, Deep learning, Human activity recognition, Pattern recognition, Sliding windows, Motion capture

## Abstract

Deep learning (DL) models are very useful for human activity recognition (HAR); these methods present better accuracy for HAR when compared to traditional, among other advantages. DL learns from unlabeled data and extracts features from raw data, as for the case of time-series acceleration. Sliding windows is a feature extraction technique. When used for preprocessing time-series data, it provides an improvement in accuracy, latency, and cost of processing. The time and cost of preprocessing can be beneficial especially if the window size is small, but how small can this window be to keep good accuracy? The objective of this research was to analyze the performance of four DL models: a simple deep neural network (DNN); a convolutional neural network (CNN); a long short-term memory network (LSTM); and a hybrid model (CNN-LSTM), when variating the sliding window size using fixed overlapped windows to identify an optimal window size for HAR. We compare the effects in two acceleration sources’: wearable inertial measurement unit sensors (IMU) and motion caption systems (MOCAP). Moreover, short sliding windows of sizes 5, 10, 15, 20, and 25 frames to long ones of sizes 50, 75, 100, and 200 frames were compared. The models were fed using raw acceleration data acquired in experimental conditions for three activities: walking, sit-to-stand, and squatting. Results show that the most optimal window is from 20–25 frames (0.20–0.25s) for both sources, providing an accuracy of 99,07% and F1-score of 87,08% in the (CNN-LSTM) using the wearable sensors data, and accuracy of 98,8% and F1-score of 82,80% using MOCAP data; similar accurate results were obtained with the LSTM model. There is almost no difference in accuracy in larger frames (100, 200). However, smaller windows present a decrease in the F1-score. In regard to inference time, data with a sliding window of 20 frames can be preprocessed around 4x (LSTM) and 2x (CNN-LSTM) times faster than data using 100 frames.

## Introduction

HAR is used in a large and ever-growing number of applications (Ramanujam, Perumal & Padmavathi, 2021). HAR ranges from gesture and pattern recognition to motion activity by analyzing the discrete measurements from different types of sensors (*e.g.*, wearable sensors, video surveillance, motion caption system (MOCAP)). This recognition can be achieved by implementing either machine learning (ML) or deep learning (DL) models (Caldas et al., 2017). With the use of wearable sensors input, DL and ML models have been implemented into several health fields of applications where real-time response is needed. However, DL-based solutions have presented more advantages than ML-based solutions in the engineering feature process, in the recognition of temporal or dynamic features and its high performance (Ramanujam, Perumal & Padmavathi, 2021). These advantages can benefit applications such as the detection of falls occurrence, an important approach in the ML classification methods (Tripathi, Jalal & Agrawal, 2018). Therefore, DL-based methodologies have been successfully implemented for fall detection and its relation with other human activities and pose recognition, using long short-term memory network (LSTM) (Chen et al., 2016; Musci et al., 2021), convolutional neural network (CNN), deep neural network (DNN) and recurrent neural network (RNN) (Hammerla, Halloran & Plötz, 2016), and other DL-based methods (Wu et al., 2022). In DL- and ML-based methods, classification is used to predict a fall from an input sequence of body postures (Hu et al., 2018). As the fall is detected the scope increments including other needs to be treated using human activity recognition (HAR). Those oriented to sports (Zhuang & Xue, 2019) and rehabilitation (Panwar et al., 2019; Xing et al., 2020); as well as degenerative diseases that involve loss of mobility such as Parkinson’s disease and knee osteoarthritis (Slade et al., 2021; Tan et al., 2021), and in assisted living which presents solutions for elderly people and also for people with visual impairments (Reyes Leiva et al., 2021).

Most HAR methodologies consist of four stages: data acquisition, data pre-processing, feature extraction, and activity classification (Caldas et al., 2017). When using wearable sensors as input, related works (Hirawat, Taterh & Sharma, 2021; Mohd Noorab, Salcic & Wang, 2017) recommend that segmentation (a procedure used to divide data measurements into smaller fragments, *i.e.*, sliding windows) be performed at the feature extraction stage. Feature extraction is a vital part of the HAR process. It helps to identify lower sets of features (factors) from input sensor data, reduces classification errors, and reduces computational complexity (Nweke et al., 2018). Depending on the AI architecture chosen to perform the classification, these fragments of data have to be processed to extract the features that feed a classifier if using ML or pass directly as input of a DL model to classify activities (Banos et al., 2014). This occurs because the feature extraction as segmentation is a conventional feature learning approach, not a DL approach since the literature suggests that data pre-processing is not compulsory in deep learning features to obtain improved results (Hammerla, Halloran & Plötz, 2016; Nweke et al., 2018). However, the window size variation has been discussed widely for being a key to see how the data size and time span affects recognition results. A large window size might include information of multiple activities, and result in an increment of computation load due to the decrease in the reactivity of the recognition system. Otherwise, with a small window size, some activities might be split into multiple consecutive windows, and the recognition task will be activated too often, without achieving high recognition results (Ma et al., 2020). Considering the discrete sensor’s measurements as time series, a sliding window set is a way to restructure a time series dataset as a supervised learning problem. Once restructured, the data works as an input to the artificial intelligence model.

The window size variation is not a common approach to evaluate accuracy for HAR methodologies based on DL using acceleration and other inertial sensor input. It is a more common approach in conventional ML methods, but some authors define a window size segmentation as pre-processing of input for HAR with DL methods. Mairittha, Mairittha & Inoue (2021) performed data labeling for an activity recognition system using inertial (acceleration and angular velocity) mobile sensing in both simple-LSTM and hybrid CNN-LSTM using 5.12s window size (at 20 Hz). With overlapping, this window size represents around 100 frames. On the other hand, Ebner et al. (2020) proposed a novel approach based on analytical transformations combined with artificially constructed sensor channels for activity recognition (acceleration and angular velocity). For this, the authors use 2, 2.5, and 3s window size at 50 Hz, which represents of 100, 125 and 150 frames, without overlapping, concluding that the differences in accuracy from window length were hardly noticeable, with a very light tendency of a decreasing accuracy with higher widths. A study of the evaluation of the window size impact on HAR for 33 fitness activities was found (Banos et al., 2014). The authors tested different window sizes ranging from 0.25 s to 7 s in steps of 0.25 s (not sampling rate defined) using four conventional classification models. They concluded that the interval 1–2 s provides the best trade-off between recognition speed and accuracy. Later, the same authors proposed the use of simultaneous multiple window sizes with a novel multiwindow fusion technique (Hu et al., 2018). Other authors experimented with the dynamic window size approach (Niazi, 2017; Ortiz Laguna, Olaya & Borrajo, 2011). As Baños et al., (2015) mentioned, no clear consensus exists on which window size should be preferably employed for HAR. If decreasing the window size allows a faster activity detection but a small window size can rely on classification errors, how small can a window be in order to keep the advantages of decreasing the window size and maintain accuracy to improve the activity recognition, and therefore the applications that require these vantages?

Deep learning models manage the feature extraction automatically, letting the computer construct complex concepts from simpler concepts (Dhillon, Chandni & Kushwaha, 2018). Therefore, it is not necessary to perform feature engineering before training the model, and as a result, the data preparation becomes more straightforward. Deep learning models can be classified into three types (Moreira, 2021): deep generative models, deep discriminative models, and deep hybrid models. Deep generative models aim to learn useful representations of data via unsupervised learning or to learn the joint probability distribution of data and their associated classes. Discriminative models learn the conditional probability distribution of classes on the data, in which the label information is available directly or indirectly. CNN and RNN are examples of this type of model. Finally, deep hybrid models combine a generative model and a discriminative model where the outcome of the generative model is often used as the input to the discriminative model for classification or regression (Funquiang Gu et al., 2021). For the current HAR experiment, four deep learning architectures, grouped by three deep discriminative and one deep hybrid model were considered: a simple DNN; a CNN; a LSTM; and a hybrid model made of a combination of a CNN-LSTM.

Although a simple DNN manages time-series data without using sliding windows, this study includes the creation of sliding windows to give an equal input setup to each AI architecture. CNN and LSTM models require an additional step to convert the problem into a supervised learning problem, as the input data must have a three-dimensional structure instead of a two-dimensional format that raw inertial sensors and motion capture system data have (Brownlee, 2018). Also, DNN models create a mapping between the inputs and class outputs of time-series data doing the feature engineering automatically (Yang, Chen & Yang, 2019). Therefore, the novelty of this research is the combination of both: automatic feature extraction process performed by DL architectures and sliding window creation as pre-processing segmentation data in order to find an optimal window size that provides the most accurate activity classification.

In the present study, three HAR activities have been considered: walking, sit-to-stand, and squatting. The two firsts have been indicated to relate to most fall studies (Zia et al., 2021); meanwhile, the squatting movement has been selected to test if the algorithm can differentiate between similar movements (sit-to-stand). As a whole, they are examples of simple human activities that serve us as a sort of laboratory to study how small can be sliding windows to keep good performance. This would be an important step to face further research on more complex HAR problems for which our results might be applicable, as might be the capability to distinguish between correct and wrong movements for a given person or to monitor the evolution of the performance when repeating a given activity. As for analyzing the effect of the sliding window size on the AI architecture performance larger and shorter sliding windows, different in about one order of magnitude, were used: 200, 100, 75, and 50 frames fixed overlapped sliding windows and 5, 10, 15, 20, 25 also overlapped frames. They represent 2, 1, 0.75, 0.50 and 0.05, 0.10, 0.15, 0.20, 0.25 s respectively, using a sampling frequency of 100 Hz. Besides, this study aims to find the optimal window size to reduce latency and the processing cost.

## Materials & Methods

### Experiment setup & data pre-processing

To evaluate the performance of the proposed methodology in the recognition of human activities, this was applied in the study of three very common daily movements, namely gait, sit-to-stand, and squatting, for a population of 10 healthy subjects. The experimental dataset was acquired at the Lisbon Biomechanics Laboratory using two systems, an inertial measurement system (IMU) (MBIENTLAB, Inc., San Francisco, CA., USA) and a high-end optoelectronic system that includes 14 infrared ProReflex 1000 cameras (Qualisys, Göteborg, Sweden) using a sampling frequency of 100 Hz. Before the acquisition, each volunteer provided his agreement by signing the informed consent after a detailed explanation of the study objectives and experimental protocol, previously approved by the ethics committee of Instituto Superior Técnico (Ref. nr. 1/2020 (CE-IST)).

The three selected movements mentioned above were recorded for 60 s using a frequency of 100 Hz. For gait the volunteers walked barefoot with self-selected cadence. In the sit-to-stand movement the volunteers were seated in a chair without a specific instruction; after a few seconds the volunteers stand up also without specific instructions to increase the variability of movement. For squatting, the volunteers performed the movement until they reached approximately 90 degrees of knee flexion and then returned to the standing position with the arms in 90 degrees flexion. The volunteers performed a series of all the movements for 60 s.

The experimental dataset consisted of 304,135 samples for the IMU system and 315,334 for MOCAP and included three-axis acceleration for each sensor and marker placed in the left ankle (lateral malleolus) and left wrist (posterior region), and a label indicating the performed activity. The final dataset has six entries or features (x1, y1, z1, x2, y2, z2). The label contained the three classes of expected activity (walking, sit-to-stand, squatting).

**Figure 1:**
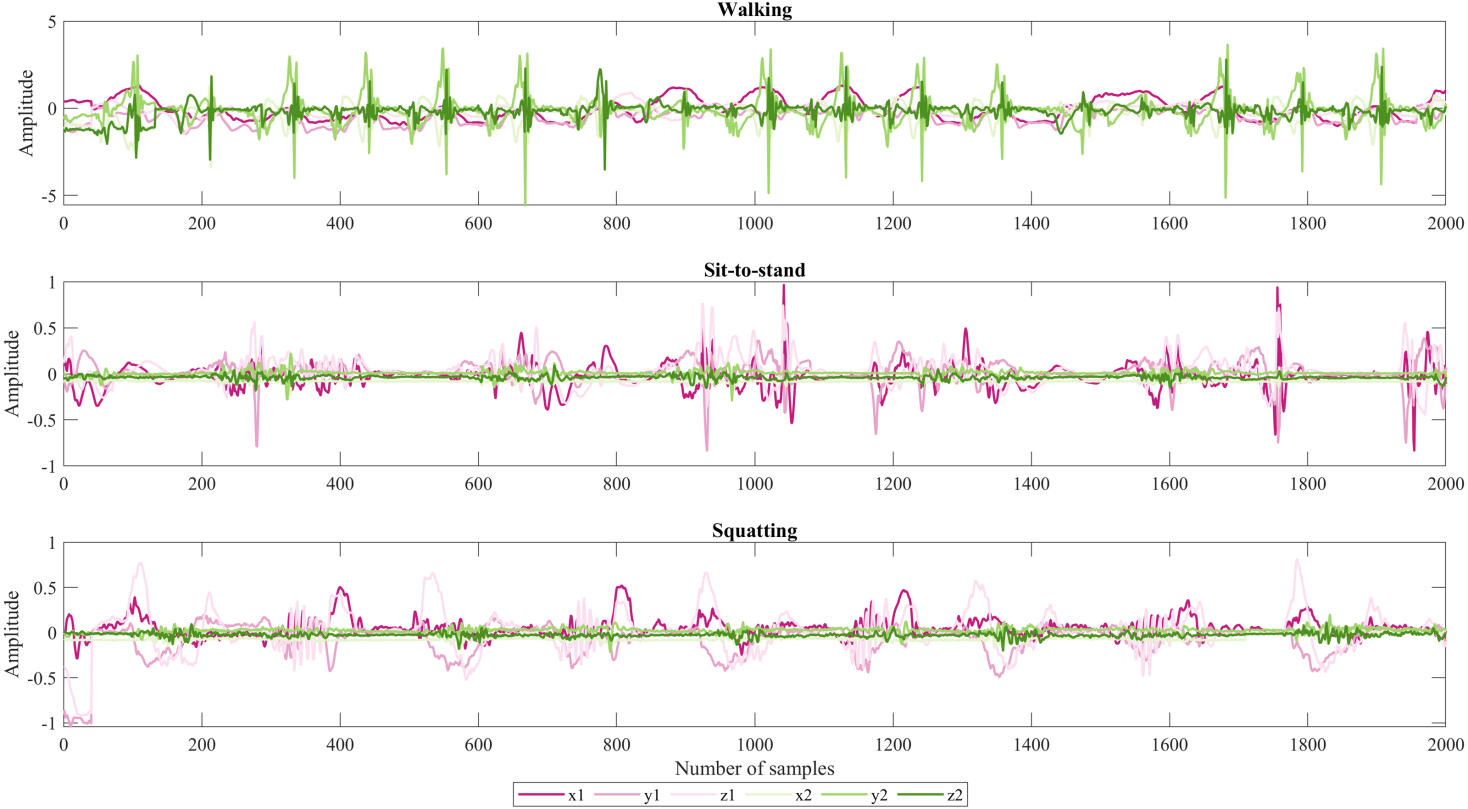
Representative set of IMU signals from participant 1.

As shown in Fig. 1. From top to bottom are the signals that correspond to accelerations in x, y, and *z* for each IMU sensor. The first three belong to the sensor that was placed in the arm and the second to the ankle, respectively. Due to sit-to-stand being similar movements the acceleration in y2 which is notorious in green color for walking is not oscillating in the two last activities because to do those activities the participant stayed almost static.

Considering that the data to evaluate the deep learning models needs to be split in training and testing, two databases per system were created: one for training with the raw data of eight volunteers and the other, with two different volunteers, for making the predictions. Hence, the IMU dataset comprised a total of 281,161 training samples and 99,108 validation samples; and the MOCAP dataset included 247,804 and 67,530 samples for training and validation respectively.

A detailed explanation of the experimental setup, marker set protocol, and pre-processing steps can be consulted in (Jaén-Vargas, 2021).

#### Sliding windows creation

There are two main ways to create sliding windows, those based on dividing the sequence following a time in seconds and those based on working with the samples (frames) of the sequence. Moreover, windows can be classified into two types: fixed or adaptive; and overlapping and non-overlapping. Windows are defined as fixed when they have the same size during the whole sequence; on another hand, they are adaptive if their size changes depending on special criteria when a movement occurs. Also, windows are overlapped when the next window stays as part of the last sequence, in other words, an overlap occurs between adjacent windows; on the contrary, it is named non-overlapping windows (Ma et al., 2020; Dehghani et al., 2019).

**Figure 2:**
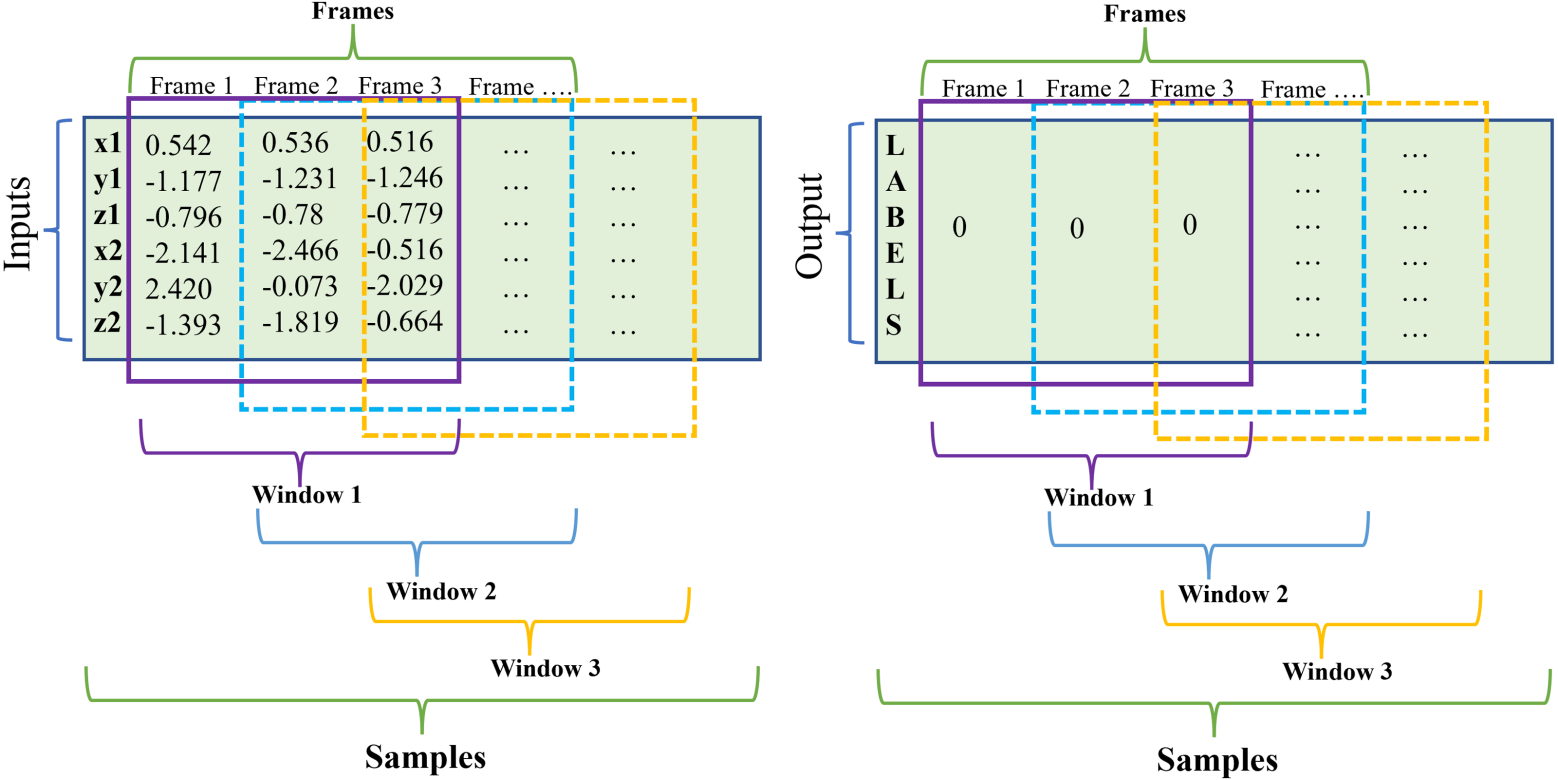
Sliding window schematic.

A sliding window comprises three elements: samples, number of time steps (window size), and features (inputs). First, a sequence is a broader sample in which there may be one or more singular samples; second, time steps or what we denominated “window size” corresponds to one observation in the sequence which may be included one or more frames; and third, a feature is the input of the sequence, in this case: x1, y1, z1, x2, y2, z2 and corresponds to one observation as a time step (see Fig. 2). When the window slides throughout the samples, a new phase is created.

In this study, the implementation of fixed overlapped sliding windows has been done and is based on dividing all the samples in a window of size *n*. In particular, the dataset was acquired with a sample rate of 100 Hz, with the first implemented window presenting a duration in seconds of 1s, thus having a size of 100 frames. This approach has a reduction in the window size (25, 20, 15, 10, 5 frames) or in other words, the time length decreases (0.25, 0.20, 0.15, 0.10, 0.05s) in order to find the optimal window parameters.

For instance, to create a sliding window of size 5, the total number of samples is divided by the timesteps, which include all the columns involved in the observation, resulting in an input shape of (281156, 5, 6). As shown in Table 1, as the window size increases, the total of samples decreases because the size of the window is subtracted from the total. In other words, a greater number of samples per window size is observed when using a larger window that slides from the beginning to the end of the time series.

**Table 1:** IMU and MOCAP window sizes’ distribution for 8 people for training the model.

**System**	**Total number of samples (8 subjects)**	**5**	**10**	**15**	**20**	**25**
IMU	281161	(281156, 5, 6)	(281151, 10, 6)	(281146, 15, 6)	(281141, 20, 6)	(281136, 25, 6)
MOCAP	247804	(247799, 5, 6)	(247794, 10, 6)	(247789, 15, 6)	(247784, 20, 6)	(247779, 25, 6)

**System**	**Total number of samples (8 subjects)**	**50**	**75**	**100**	**200**
IMU	281161	(281111, 50, 6)	(281086, 75, 6)	(281061, 100, 6)	(280961, 200, 6)
MOCAP	247804	(247754, 50, 6)	(247729, 75, 6)	(247704, 100, 6)	(247604, 200, 6)

**Notes.**

1 Window sizes for IMU and MOCAP.

Therefore, in this study, two sets of different windows have been created: one of shorter lengths (5, 10, 15, 20, and 25 frames), and the other of longer lengths (50, 75, 100, and 200 frames), in order to compare the performance metrics (accuracy and F1-score) that are achieved for each tested architecture of deep learning, thus allowing us to find the window size that would be optimal to reduce the cost of processing.

### Artificial intelligence models

These architectures were planned from simple to robust to see their performances varying in the two sets of sliding windows mentioned above. The parameters were set as shown in Table 2.

**Table 2:** Parameters of each deep learning architecture.

**Parameters**	**DNN**	**CNN**	**LSTM**	**CNN-LSTM**
Layers with Neurons	3 Dense (32 neurons), Flatten, SoftMax	1D-Conv (32 neurons, filter=3 kernel=3), MaxPooling (size=2), Flatten, Dense (8 neurons), SoftMax	LSTM (100 neurons), Flatten, Dense (100 neurons), SoftMax	2 1D-Conv (64 neurons, filter=3 kernel=3), MaxPooling (size=2), Flatten, LSTM (100 neurons), Flatten, Dense (100 neurons), SoftMax
Dropout rate	0	0.5	0.5	0.5
Activation function	ReLU	ReLU	ReLU	ReLU
Optimizer	Adam	Adam	Adam	Adam
Loss function	Sparse categorical crossentropy	Sparse categorical crossentropy	Sparse categorical crossentropy	Sparse categorical crossentropy
Batch size	64	32	64	64
Epochs	100	100	100	100

**Notes.**

2 Software: Python, Tensor Flow, Google Colab.

**Figure 3:**
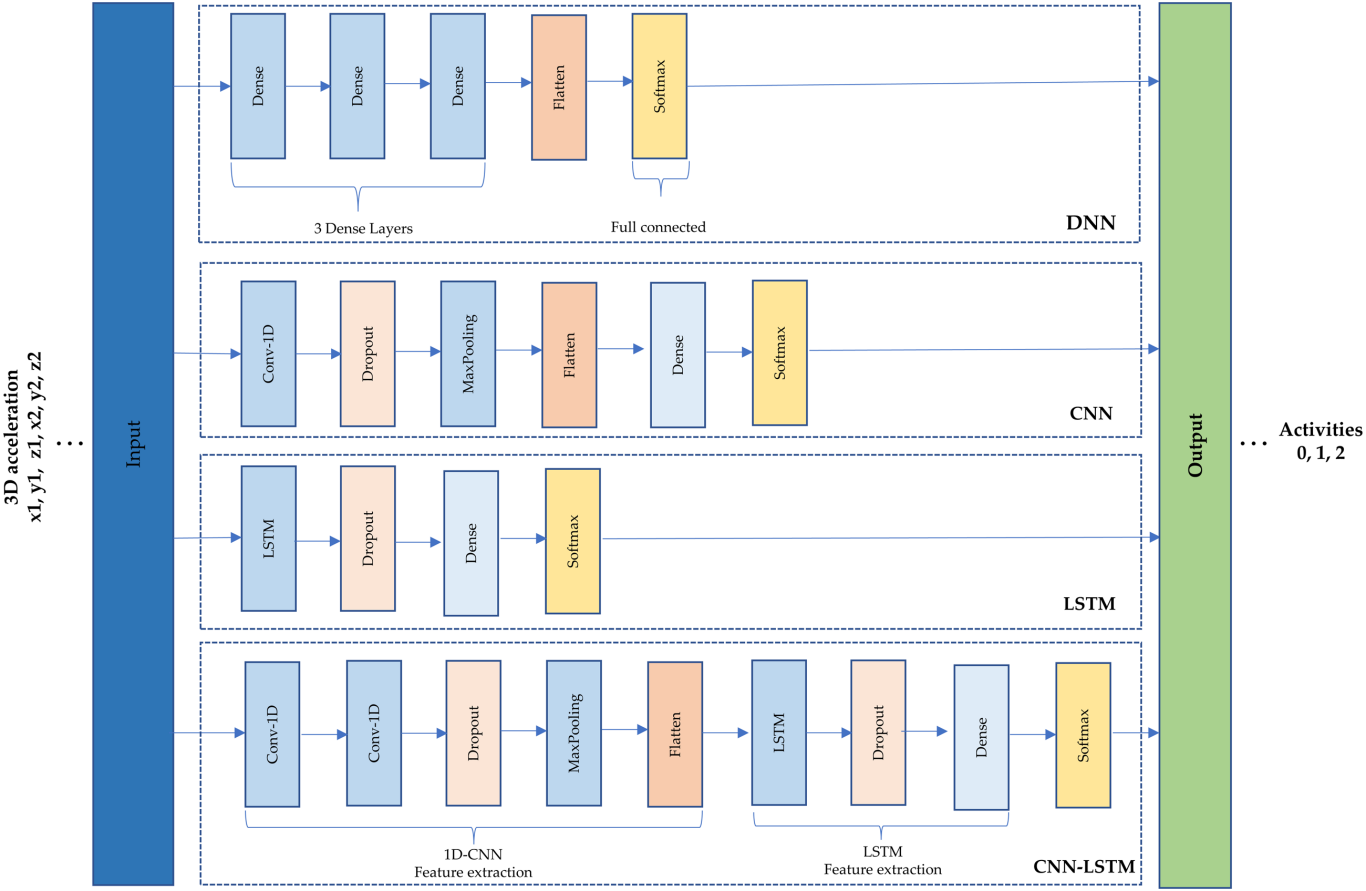
(1) Deep neural network architecture. (2) Convolutional neural network architecture. (3) Long short-term memory architecture. (4) Hybrid model (CNN-LSTM) architecture.

A schematic of the four architectures is presented in Fig. 3. First, a simple DNN was implemented using fully connected layers. Three dense layers of 32 neurons were set, a flatten layer and a SoftMax were used to get the output (see Fig. 3(1)). Second, CNN is based on convolutions to capture dependencies among input data (Murad & Pyun, 2017). A one-dimensional convolutional neural network (1D-CNN) was used within a filter and kernel of size three; a dropout of 0.5 to minimize overfitting, a max-pooling layer of size two, a flatten and a dense layer of eight neurons, and a SoftMax (see Fig. 3(2)). This type of architecture is recommended to learn detailed feature representations and patterns from images (Gollapudi, 2019) but the 1D was used because the data is time series.

Third, LSTM is the architecture most often recommended to treat time series data because it is the type of recurrent neural network (RNN) used to train the model over lengthy sequences of data. Due to this, it aims to retain the memory from previous time steps to feed the model (Goyal, Pandey & Jain, 2018). An LSTM architecture was used that comprises 100 neurons, a dropout of 0.5, a dense layer of 100 neurons, the SoftMax to obtain the output as shown in Fig. 3(3)

Finally, a hybrid model named CNN-LSTM was chosen as the last architecture. This model reads subsequences of the main sequence as blocks: CNN extracts features from each block, and then allows the LSTM to interpret the features extracted from each block. For this, a time distributed wrapper is needed to allow reusing of the CNN model, once per each subsequence, and the CNN output serves as input to the LSTM, which provides the final prediction. In other words, this hybrid model uses CNN layers for feature extraction on input data and LSTMs to support sequence prediction (Yao et al., 2017). As shown in Fig. 3(4), the hybrid model comprises a 1D-CNN with five layers and an LSTM with four layers.

### Performance metrics and evaluation

The performance is measured using the loss function sparse categorical cross-entropy. This function is used when there are two or more label classes which are integers. In our case, there are three labels or classes provided (0, 1, 2) for walking, sit-to-stand, and squatting. Also, as the activation function rectified linear unit activation (ReLU) was chosen. Regarding optimizers, the Adam algorithm was used which is a stochastic gradient descent method that is based on adaptive estimation of first-order and second-order moments.

To evaluate the classification performance of each deep learning model two metrics were chosen: accuracy which corresponds to the ratio of the number of correct predictions to the total number of input samples (Mishra, 2018); and the F1-score that combines two measures defined in terms of the total number of correctly recognized samples, which are known as precision and recall (Ordóñez & Roggen, 2016). A higher accuracy or F1-score value implies a better performance in the model (Yan et al., 2020).

**Figure 4:**
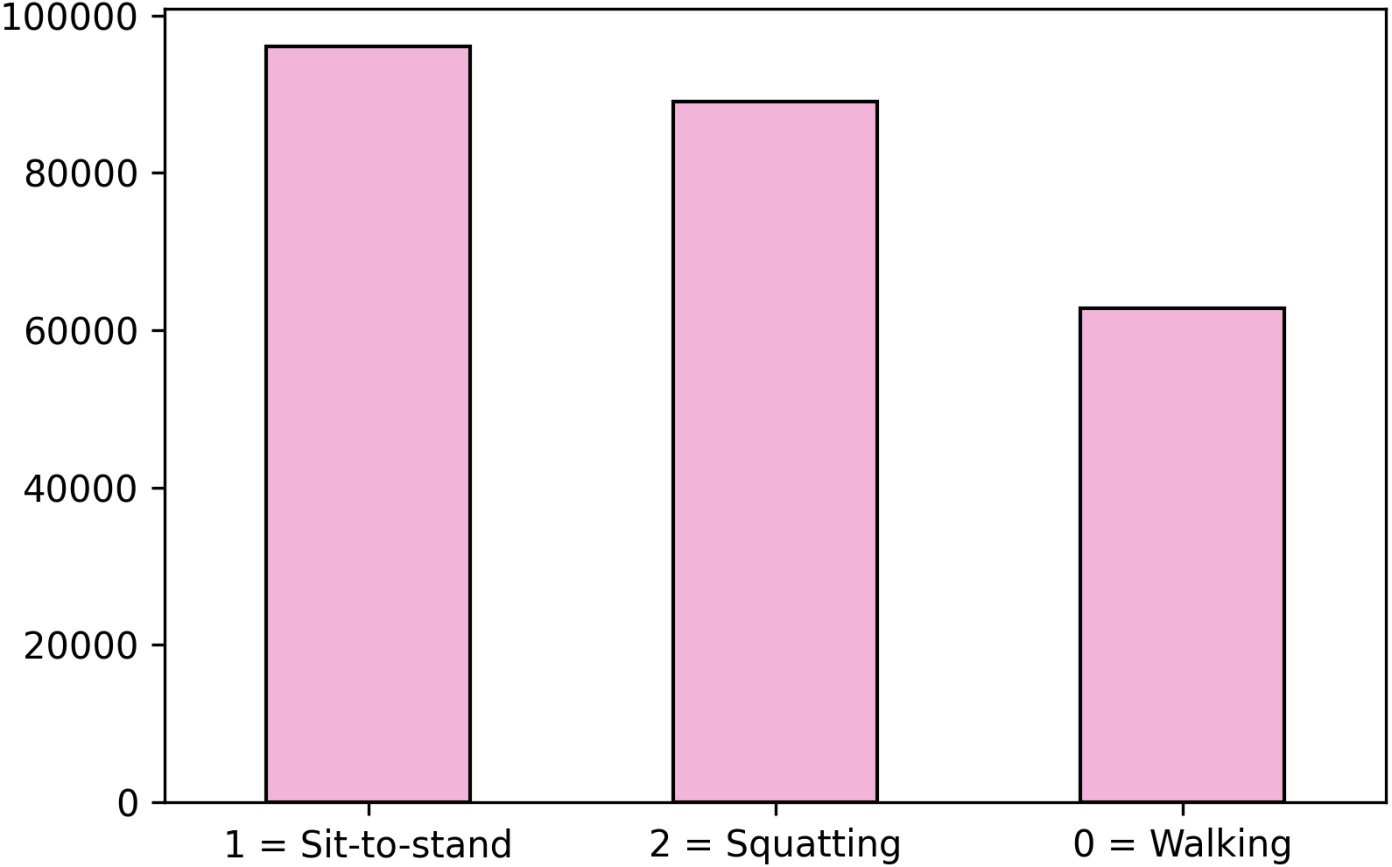
Class 0 is not equally balanced.

Despite accuracy is the conventional evaluation metric when problems have no class imbalance (He & Garcia, 2019), another parameter that faces this problem needs to be included due to this study presents class imbalance for MOCAP (see Fig. 4). For this, the F1-score is considered which counts the class imbalanced by weighting the classes according to their sample proportion. Due to constraints with the volume of acquisition of the MOCAP system, the dataset for this system is not fully balanced. In particular, the number of frames for the walking movement is 35% less than the big one as shown in Fig. 4. For this, the F1-score is considered which counts the class imbalance by weighting the classes according to their sample proportion.

In addition, DL models were also evaluated based on the inference time (the response time of the model). The best inference time is indicated for instance, if a risk situation, the system response time should be as low as possible (Sarabia-Jácome et al., 2020).

## Results

As mentioned in the Introduction section, the main focus of this study was to find which is the most appropriate window size to obtain acceptable performance metrics with a low inference time. First, a window size of 100 was tested considering the same frequency rate that was used to record the three activities. Second, the window size was doubled (200 frames) and after that, the window size decreased using 75 and 50 samples. Due to there were not relevant changes in accuracy and the F1-score (see Table 3) using the long ones, a reduction in window size was registered using shorter (25, 20, 15, 10, 5).

**Table 3:** Performance metrics in window sizes of 200, 100, 75, and 50 for IMU and MOCAP.

**Model**	**IMU 200**		**IMU 100**		**IMU 75**		**IMU 50**	
	**Accuracy**	**F1-score**	**Accuracy**	**F1-score**	**Accuracy**	**F1-score**	**Accuracy**	**F1- score**
DNN	0.99	95.64	0.99	91.85	0.99	89.38	0.98	88.41
CNN	0.99	94.91	0.98	91.35	0.97	92.66	0.94	90.36
LSTM	0.94	95.95	0.99	91.85	0.99	92.34	0.99	88.79
CNN-LSTM	0.99	96.84	0.99	99.97	0.99	91.17	0.99	90.82

**Model**	**MOCAP 200**		**MOCAP 100**		**MOCAP 75**		**MOCAP 50**	
	**Accuracy**	**F1-score**	**Accuracy**	**F1-score**	**Accuracy**	**F1-score**	**Accuracy**	**F1-score**
DNN	1.00	89.07	0.99	87.19	0.99	86.43	0.99	83.89
CNN	0.99	89.49	0.98	86.30	0.98	88.60	0.96	87.57
LSTM	0.99	89.75	0.99	87.83	1.00	88.08	0.99	85.96
CNN-LSTM	0.99	89.89	0.99	88.63	0.99	88.14	0.99	86.20

For IMU data, the accuracy obtained surpasses 90.08% using a window of 20 frames in advance, as shown in Table 4. Moreover, this result is better using the two last architectures: the LSTM and the hybrid model, which both achieve 99%. On the other hand, for the F1-score, an increment is observed using a window of 20 frames which is around 83.35%. For the simplest networks (DNN and CNN) as the size of the window is smaller (5, 10, 15) accuracy and F1-score are not high enough which would affect final predictions.

**Table 4:** Performance metrics in window sizes of 5, 10, 15, 20, and 25 for IMU and MOCAP.

**Model**	**IMU 5**		**IMU 10**		**IMU 15**		**IMU 20**		**IMU 25**	
	**Accuracy**	**F1-score**	**Accuracy**	**F1-score**	**Accuracy**	**F1-score**	**Accuracy**	**F1-score**	**Accuracy**	**F1-score**
DNN	0.86	79.53	0.89	80.70	0.91	81.68	0.94	83.36	0.96	82.72
CNN	0.82	79.74	0.87	83.59	0.89	84.76	0.90	88.01	0.92	88.87
LSTM	0.91	80.80	0.96	82.22	0.99	83.47	1.00	85.18	1.00	86.93
CNN-LSTM	0.89	83.56	0.95	84.57	0.98	85.40	0.99	87.08	1.00	87.50

**Model**	**MOCAP 5**		**MOCAP 10**		**MOCAP 15**		**MOCAP 20**		**MOCAP 25**	
	**Accuracy**	**F1- score**	**Accuracy**	**F1- score**	**Accuracy**	**F1- score**	**Accuracy**	**F1- score**	**Accuracy**	**F1- score**
DNN	0.86	77.73	0.89	77.64	0.94	78.75	0.94	78.31	0.96	81.19
CNN	0.81	76.19	0.87	79.56	0.89	81.70	0.92	82.74	0.93	83.67
LSTM	0.93	74.60	0.96	77.48	0.99	78.60	1.00	80.57	1.00	82.21
CNN-LSTM	0.89	79.94	0.95	80.06	0.97	81.37	0.99	82.80	0.99	84.36

In regard to MOCAP data (see Table 4), accuracy is above 87% using windows from 10 frames to 25. Being 20 frames, which achieves 99% for LSTM and 98.88% for CNN-LSTM. For the F1-score, the window which comprises the 20 frames results in 82.80% using the CNN-LSTM model. The sliding windows smaller than 20 (15, 10, 5) have an F1-score below 80%, therefore the prediction will be affected and won’t have high accuracy.

Moreover, Table 5 illustrates the precision, recall, and F1-score metrics values for the four deep learning algorithms corresponding to each window size. As a result, a window of 20 frames presents a precision between 85 and 87%.

**Table 5:** Recall and precision for IMU and MOCAP.

**Model**	**IMU 5**		**IMU 10**		**IMU 15**		**IMU 20**		**IMU 25**	
	**Precision**	**Recall**	**Precision**	**Recall**	**Precision**	**Recall**	**Precision**	**Recall**	**Precision**	**Recall**
DNN	80.56	79.14	81.62	80.51	82.45	81.60	83.45	83.27	83.07	82.55
CNN	80.73	79.37	84.27	83.28	85.79	84.63	85.18	87.81	89.26	88.69
LSTM	81.08	80.60	82.30	82.16	83.89	83.67	85.17	85.19	87.00	86.88
CNN-LSTM	83.80	83.45	84.82	84.48	85.47	85.19	87.23	87.83	87.64	87.44

**Model**	**MOCAP 5**		**MOCAP 10**		**MOCAP 15**		**MOCAP 20**		**MOCAP 25**	
	**Precision**	**Recall**	**Precision**	**Recall**	**Precision**	**Recall**	**Precision**	**Recall**	**Precision**	**Recall**
DNN	78,40	77.40	79.89	77.32	80.50	78.44	81.53	78.18	83.03	81.01
CNN	78.23	75.73	81.40	79.04	82.50	81.36	84.50	82.34	85.24	83.32
LSTM	76.56	74.23	79.78	77.15	81.41	78.45	83.56	80.29	84.62	82.06
CNN-LSTM	81.25	79.56	82.21	79.74	83.38	81.08	84.55	82.57	85.75	84.14

**Model**	**IMU 200**		**IMU 100**		**IMU 75**		**IMU 50**	
	**Precision**	**Recall**	**Precision**	**Recall**	**Precision**	**Recall**	**Precision**	**Recall**
DNN	95.68	95.64	91.84	91.86	89.67	89.29	88.46	88.43
CNN	94.90	94.92	91.43	91.34	92.68	92.66	90.53	90.32
LSTM	96.14	95.93	91.84	91.86	92.44	92.32	88.84	88.76
CNN-LSTM	96.84	96.84	99.97	99.97	91.20	91.15	90.98	90.75

**Model**	**MOCAP 200**		**MOCAP 100**		**MOCAP 75**		**MOCAP 50**	
	**Precision**	**Recall**	**Precision**	**Recall**	**Precision**	**Recall**	**Precision**	**Recall**
DNN	90.93	88.92	89.25	87.01	88.60	86.26	86.15	83.73
CNN	91.38	89.31	89.45	86.07	89.94	88.46	88.73	87.34
LSTM	92.05	89.68	90.28	87.61	89.96	87.89	87.96	85.80
CNN-LSTM	91.57	89.70	90.49	88.41	89.92	82.21	88.41	85.98

Furthermore, Table 6 presents the sensitivity and specificity of the four DL architectures tests. As is shown in Table 6, the window of 20 frames presents a sensitivity between 83 and 87%, and specificity from 91 to 93%.

**Table 6:** Sensitivity and specificity for IMU and MOCAP.

**Model**	**IMU 5**		**IMU 10**		**IMU 15**		**IMU 20**		**IMU 25**	
	**Sensitivity**	**Specificity**	**Sensitivity**	**Specificity**	**Sensitivity**	**Specificity**	**Sensitivity**	**Specificity**	**Sensitivity**	**Specificity**
DNN	79.14	89.57	80.52	90.26	81.60	90.80	83.27	91.64	82.56	91.28
CNN	79.37	89.69	83.28	91.64	84.63	92.31	87.81	93.91	88.70	94.35
LSTM	80.61	90.30	82.16	91.08	83.67	91.83	85.19	92.60	86.89	93.44
CNN-LSTM	83.46	91.73	84.48	92.24	85.19	92.60	87.03	93.52	87.44	93.72

**Model**	**MOCAP 5**		**MOCAP 10**		**MOCAP 15**		**MOCAP 20**		**MOCAP 25**	
	**Sensitivity**	**Specificity**	**Sensitivity**	**Specificity**	**Sensitivity**	**Specificity**	**Sensitivity**	**Specificity**	**Sensitivity**	**Specificity**
DNN	77.40	88.70	77.32	88.66	79.06	89.53	78.18	89.09	81.01	90.51
CNN	75.73	87.87	79.04	89.52	79.04	89.52	82.34	91.17	79.04	89.52
LSTM	74.23	87.11	77.15	88.58	78.45	89.22	80.29	90.15	77.15	88.58
CNN-LSTM	79.56	89.78	79.74	89.87	81.08	90.54	82.57	91.28	79.74	89.87

**Model**	**IMU 200**		**IMU 100**		**IMU 75**		**IMU 50**	
	**Sensitivity**	**Specificity**	**Sensitivity**	**Specificity**	**Sensitivity**	**Specificity**	**Sensitivity**	**Specificity**
DNN	95.64	97.82	91.87	95.93	89.29	94.65	88.43	94.22
CNN	94.92	97.46	91.34	95.67	92.66	96.33	90.32	95.16
LSTM	95.93	97.96	91.87	95.93	82.16	91.08	88.76	94.38
CNN-LSTM	96.85	98.42	99.97	99.99	91.15	95.58	90.75	95.37

**Model**	**MOCAP 200**		**MOCAP 100**		**MOCAP 75**		**MOCAP 50**	
	**Sensitivity**	**Specificity**	**Sensitivity**	**Specificity**	**Sensitivity**	**Specificity**	**Sensitivity**	**Specificity**
DNN	88.92	94.46	87.01	93.51	86.26	93.13	83.73	91.86
CNN	89.31	94.66	86.07	93.04	88.46	94.23	87.34	93.67
LSTM	89.68	94.84	87.62	93.81	87.90	93.95	85.80	92.90
CNN-LSTM	89.70	94.85	88.41	94.21	87.94	93.97	85.98	92.99

**Figure 5:**
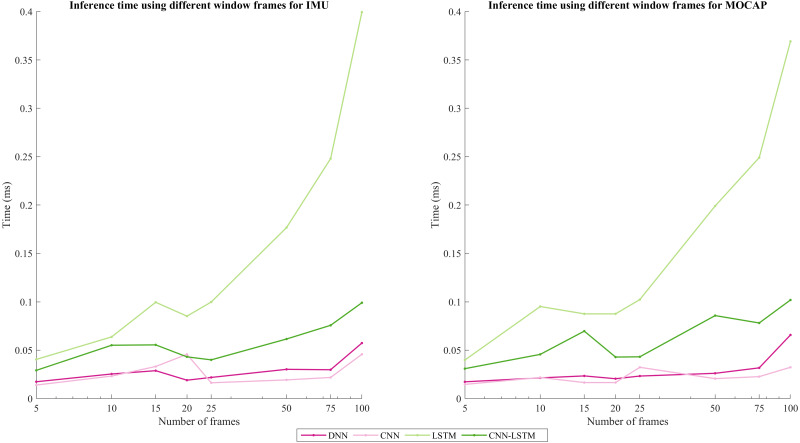
Inference time comparison using different windows frames for IMU and MOCAP.

In addition, inference time was calculated using an HP OMEN laptop with an AMD Ryzen 7 4800H processor with Radeon Graphics 2.90 GHz. Also, it counts with 16GB RAM. The inference time for all the window sizes was always under 1 ms. The LSTM model took more time: around 0.4 ms using 100 frames and below 0.1 ms for a window size of 20; followed by the hybrid model CNN-LSTM, CNN, and finally the DNN. Figure 5 shows the inference time results using the different window sizes (5, 10, 15, 20, 25, 50, 75, and 100) for each DL architecture. A disruption is observed on the window of size 15 for the LSTM model. Also, in the window of 20 frames, the CNN architecture experienced an increase too, but the rest of the architectures presented a decrease in this window size. On the other hand, for MOCAP, a disruption is observed on window size = 15 for the CNN-LSTM model, and a decrease is noted for 20 frames in all the architectures. However, a trend is maintained, which means that as the window size (the number of frames) increases, the inference time will increase.

Finally, a sliding window of 20 frames in data acquired using a sampling frequency of 100 Hz is the minimal size for obtaining a high accuracy (99% and 98% for LSTM and CNN-LSTM), acceptable F1-score (85.18% and 87.07%), and with a low inference time (below 1 ms) for HAR of 3 activities: walking, sit-to-stand, and squatting. Despite two similar movements (sit-to-stand and squatting) were selected, the neural networks were able to distinguish between them and classify them properly.

**Figure 6:**
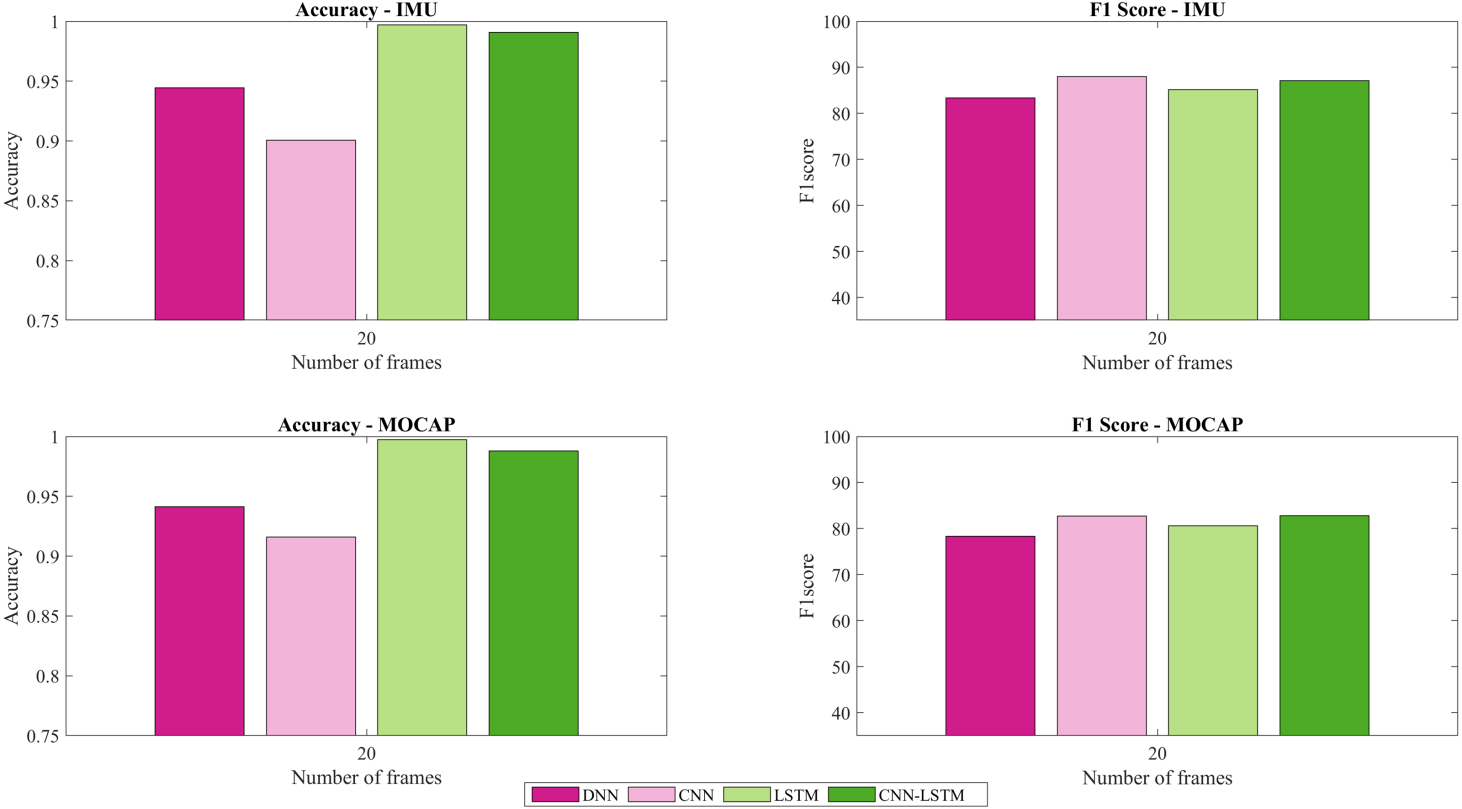
Accuracy and F1-score for IMU and MOCAP using a sliding window of size 20.

Figure 6 presents the accuracy and F1-score for IMU and MOCAP, using a sliding window of 20 frames.

**Figure 7:**
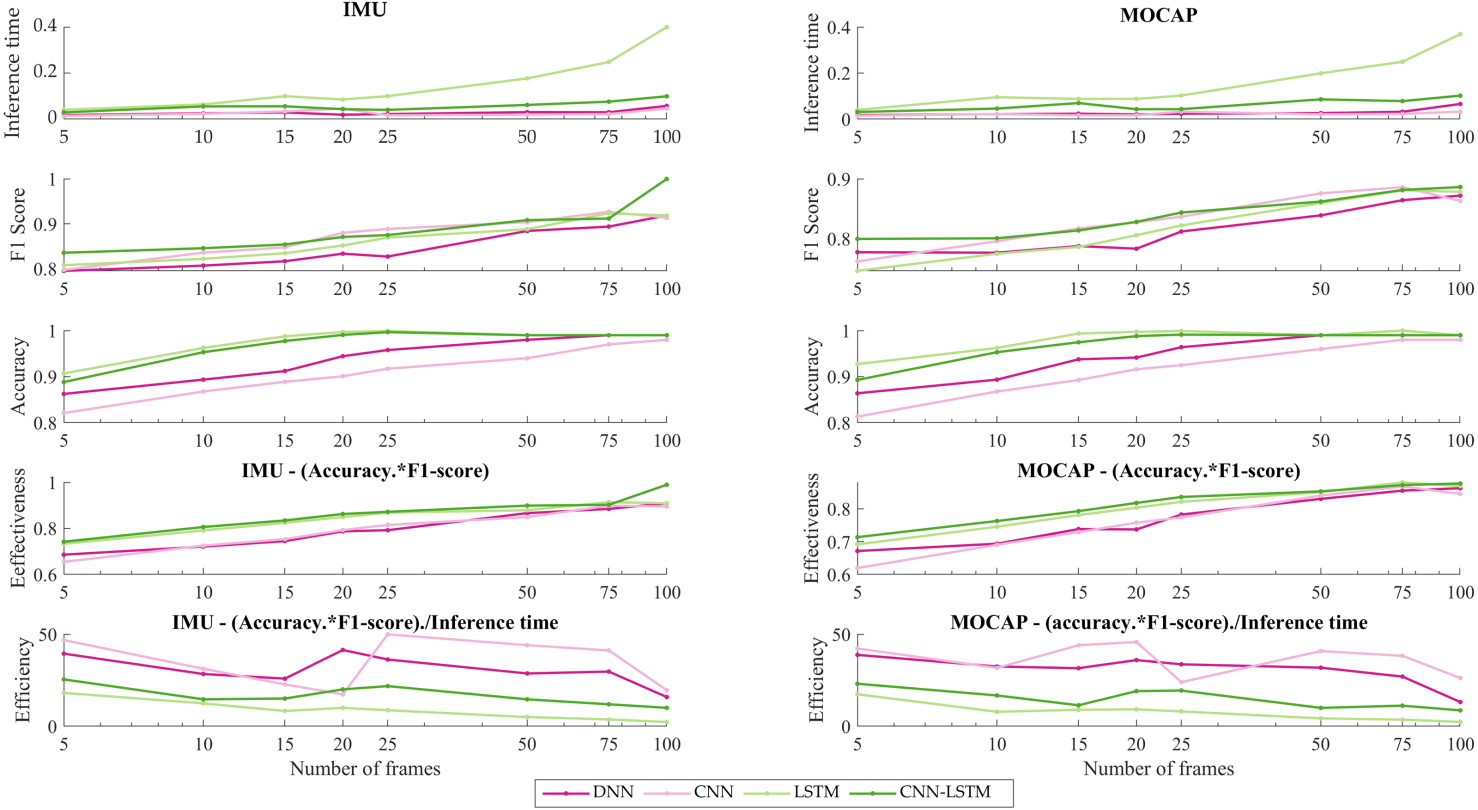
IMU vs MOCAP. (1) Inference time. (2) F1-score. (3) Accuracy. (4) Effectiveness. (5) Efficiency.

Figure 7 presents the effectiveness and efficiency of DL models varying window sizes.

## Discussion

The importance of sliding windows as a segmentation pre-processing tool in the input of a classification neural networks especially for doing HAR is mentioned in (Attal et al., 2015). Most of the studies consider the sliding window method by dividing all sequences throughout time as mentioned in the Introduction section. On the contrary, this current study considered analyzing the sequence based on frames in order to make a relation with literature’s terminology. In Wang et al. (2018), differentiate the impact of window size for motion mode recognition and pose. For motion, increasing the window size may affect the cut-off window length and sacrifice the recognition speed. They suggest as performance requirements a cut-off length of 6s with an F1-score beyond 99%. Also, if the focus is to reduce the latency, a shortened window is needed and a reduction in accuracy is expected. For instance, a window between 2.5–3.5 s with an F1-score of around 95% was suggested (Wang et al., 2018). In this current work, patterns and features have been extracted more accurately using LSTM and CNN-LSTM. Also, the indicator of performance reflects an F1-score that surpasses 80% for both types of data: IMU and MOCAP using a sliding window of 20 frames (see Fig. 6), a window which is smaller than the used in Wang et al. (2018). A reduction of cost processing was obtained when placing the trained model in production, which is in agreement with other applications (Gupta, 2021) where the proper selection of sliding windows (window size) is important to also ensure that clinical information due to subtle changes are captured from the analyzed signal.

Furthermore, in Banos et al. (2014), says that 1 to 2 s correspond to the interval which provides the best trade-off between recognition and accuracy. Thus, this current work agrees with them because the highest accuracy was found in 100 frames or 1s. Also, that work (Banos et al., 2014) analyzed the effects of window process on activity recognition performance, thus, a reduction in window size was done obtaining the most precise recognizer for very short windows being (0.25–0.5 s), the perfect interval for recognition of most activities. This study is in accordance with Banos due to 20 frames (0.20 s) corresponding to the most proper window size for the recognition of these three activities (walking, sit-to-stand, and squatting).

Regarding other approaches to preprocess the data, in Gholamiangonabadi, Kiselov & Grolinger (2020), mentioned that the features were obtained using four pre-processing scenarios: using vector magnitude, sliding windows, vector magnitude and applying sliding window to this data, and without any pre-processing. It concluded that the accuracy increased with the incrementation of the value of sliding windows from 5, 10, 15, 20, and 50. Also, there were considered other techniques for evaluating the model: cross-Validation and Leave-One-Subject-Out Cross-Validation (LOSOCV). The accuracy was incremented from 85% to 98% using LOSOCV. In contrast, in this study, a train-test split was used for the evaluation and the accuracy was similar to theirs.

Additionally, in Abbaspour et al. (2020) have shown the performance of four DL hybrid models to do HAR. As a result, the models that include Bi-directional RNNs perform better than the uni-directional RNNs. Also, regarding sensitivity, there was not huge difference observed in all the models above 93%; however, specificity reached 99% for all the hybrid models. In contrast, this current work tested CNN-LSTM uni-directional hybrid model reaching better accuracy and F1-Score. In addition, the sensitivity for a window size of 20 in IMU was 87.03% and specificity was 93.52%.

In this study, the previous work (Jaén-Vargas, 2021) that considered a sliding window of 100 frames (1s) has been completed. Performance metrics resulted in about 88% for the F1-score but this implies more processing costs and does not vary as much as the case when using a window of 20 frames enhancing an F1-score of 82.80% for MOCAP. Moreover, for IMU data the F1-score obtained was 99% using 100 frames (1s) and 83.35% using 20 frames (0.20s). As shown in Fig. 5, the window that presents a size of 20 has the best accuracy per inference time. Also, despite the third and fourth architectures (LSTM, CNN-LSTM) that imply more parameters resulted in the best solutions, since they have the best accuracy, and the time is still considerably low to be used in real-time.

As for the inference time, in Mairittha, Mairittha & Inoue (2021)) it is calculated in two of the models presented here: LSTM and CNN-LSTM. The inference times obtained were 0.0106s and 0.3941s, respectively, resulting in LSTM’s is around 3X slower than CNN-LSTM’s. On the contrary, in this current work, the inference time is in the order of ms and was higher for LSTM than the hybrid model CNN-LSTM, being in a window of 20, 0.0852 ms, and 0.0431 ms, respectively, which represents that LSTM is 2X faster than CNN-LSTM. Comparing 100 frames with 20 frames, using a window of 20 frames the LSTM is 4X and CNN-LSTM is 2X faster than in a window of 100 frames.

Furthermore, in order to discuss the better performance per inference time in this work, the effectiveness and efficiency of DL’s models varying window sizes have been calculated (see Fig. 7). As is shown in Figs. 7(4), effectiveness resulting from multiplying the performance metrics: accuracy and F1-score, is noticed that around 20 frames the accuracy surpasses 80% being LSTM as closed as CNN-LSTM architecture which this last model achieves the best accuracy for IMU. However, for MOCAP the stabilization was observed around 20 to 25 frames. Also, a disruption is reflected in 75 frames for both systems (IMU and MOCAP) due may be to this particular window size overlapping two different activities and this causes a decrease in efficacy. On another hand, in Figs. 7(5), the efficiency which implies effectiveness per inference time, for IMU, the CNN presented the highest efficiency about 57% using 25 frames but due to the inference time is low (order of milliseconds) for the four DL architectures, the hybrid model (CNN-LSTM) is chosen as the best because its stability over time. As it is the IMU’s case, for MOCAP, the CNN model presented more efficiency, but the same stabilization that is shown in IMU from 20 frames is noticeable since 25 frames for MOCAP.

At last, this work explores the applicability of DL methodologies with sliding windows to be used in HAR activities. The results obtained presented an excellent accuracy in the identification of the tested models, even considering that two similar movements were used in the learning and testing steps. Moreover, the time needed to identify the movements were quite short, since all tested configuration presented values under 1 ms. These outcomes allow the use of these methodologies in HAR in real-time. Additionally, this study shows that accelerometer data can be efficiently used in the recognition of different human movements. This issue is particularly relevant, as it can be implemented in technologies used daily, such as the mobile phone, smart-watch, or any simple device developed using an accelerometer.

As limitations, a fixed frequency was used in the data acquisition process, therefore other results may be obtained if different frequencies are considered. Moreover, the present study considers only 3 activities, so the network might be restricted. Hence, future works may consider the analysis of a larger number of movements and also the analysis of non-pathological and pathological.

Future research on more complex HAR problems for which our results might be applicable, as might be the capability to distinguish between correct and wrong movements for a given person, or to monitor the evolution of the performance when repeating a given activity. Also, a test with other frequencies to obtain the optimal window size for different systems, for instance in kinetics where depth cameras or video cameras with human tracking are used, since these systems usually work with lower frequencies.

## Conclusions

The impact of using deep learning models as well as an appropriate size for the sliding window aims in the recognition of human activities and affects the cost of processing. In this study, after comparing the results for IMU and MOCAP data, it can be concluded that it is not necessary to use big sliding windows to get high-performance metrics (good prediction) because it will increment the cost of processing (inference time) and capability of the response of any application that will create using the trained model. Hence, choosing an adequate window size and the use of deep learning algorithms to interpret the sequences comprised by the 3D acceleration of each system used are important to obtain a final prediction.

Regardless of the dataset used: IMU or MOCAP data, using the more specialized deep neural architectures (LSTM and CNN-LSTM) and a minimal overlapping window of 20 frames, lead to obtaining higher accuracy (above 90%), an F1-score (above 80%), and an inference time (below 0.1 ms) for both systems. Furthermore, with the usage of large sliding windows (100, 200 frames), there are almost no accuracy differences. However, small sliding windows present a decrease in the F1-score.

Overall, this study presents an inference time less than 1 ms for sliding windows ranging from 5 to 100 frames (0.05 to 1s) and tested for each DL architecture such as DNN, CNN, LSTM, and CNN-LSTM. Hence, data segmented using a sliding window of 20 frames can be preprocessed 4X (LSTM) and 2X (CNN-LSTM) faster than using 100 frames. Consequently, this low inference time is suggested for real-time applications, for instance, to detect given movements.

In addition, this work has been conducted using a fixed frequency of 100 Hz. Other results will be obtained if variable frequencies are used (20, 30, and 50 Hz) because sliding windows will vary.

### Institutional Review Board Statement

The study was conducted according to the guidelines of the Declaration of Helsinki and approved by the ethics committee of Instituto Superior Técnico (Ref. nr. 1/2020 (CE-IST), 10/01/2020).

## Additional Information and Declarations

### Data Availability

The following information was supplied regarding data availability: The data is available at GitHub:
https://github.com/mjaenvargas/Sliding_Window_DL_IMU_MOCAP.
